# Enhancing Probiotic Performance in Food Matrices: From Strain Selection to Bioengineering

**DOI:** 10.1111/1750-3841.71371

**Published:** 2026-08-19

**Authors:** Sidney Rodrigues de Jesus Silva, Ramila Cristiane Rodrigues, Rosângela Freitas, Paulo César Stringheta, Pedro Henrique Campelo, Evandro Martins

**Affiliations:** ^1^ Laboratory of Hygiene and Food Microbiology (LHMA), Department of Food Technology Federal University of Viçosa Viçosa Brazil; ^2^ LaCBio, Laboratory of Natural Pigments and Bioactives, Department of Food Technology Federal University of Viçosa Viçosa Brazil; ^3^ InovaLeite, Milk and Dairy Research Laboratory, Department of Food Technology Federal University of Viçosa Viçosa Brazil

## Abstract

Probiotics are central to functional food innovation, but their application is limited by viability loss during processing, storage, and gastrointestinal (GI) transit. A key insight is that viability and functionality are distinct attributes; maintaining high cell counts does not guarantee that probiotics retain their beneficial properties. Despite extensive literature on individual probiotic delivery strategies, no prior review has integrated strain selection, matrix design, protective technologies, and bioengineering into a single end‐to‐end framework. This review examines strategies to enhance probiotic performance across food matrices, emphasizing that effective delivery requires preserving both viability and functionality. Strain selection based on stress tolerance and adaptation via sublethal exposure are key approaches to improve robustness. Dairy matrices provide inherent protection to probiotics, whereas nondairy systems, including fruits, cereals, meat, and chocolate, require complementary technologies to enhance probiotic survival. Microencapsulation methods, including spray drying, extrusion, emulsification, and electrospraying, enhance probiotic stability and enable targeted GI release. Furthermore, prebiotic co‐encapsulation and synbiotic formulations improve survival and functionality through synergistic effects. Advances in genetic engineering, including CRISPR‐based tools, enable the development of next‐generation probiotics with improved stress tolerance and metabolic capabilities, although regulatory challenges remain. We conclude that effective probiotic delivery depends on integrating strain selection, matrix design, protective technologies, and bioengineering tailored to specific applications while preserving functionality throughout shelf life.

## 1 Introduction

According to market projections based on industry reports and commercial forecasts, the global nutraceutical market, valued at approximately $458.5 billion in 2024, is projected to reach approximately $986.85 billion by 2032, with a compound annual growth rate exceeding 10% (Fortune Business Insights 2025). This expansion is driven by aging populations, rising healthcare costs, and a shift toward preventive, food‐based health strategies (Santini et al. 2023). The COVID‐19 pandemic further boosted demand for dietary supplements and functional foods, more than doubling US sales at the onset and sustaining elevated consumer interest (Santini et al. 2023; Sarita et al. 2025). Within this sector, probiotics have emerged as one of the fastest‐growing categories, with the global market projected to exceed $220 billion by 2030 (Grand View Research 2024).

According to the FAO/WHO, probiotics are defined as “live microorganisms which, when administered in adequate amounts, confer a health benefit on the host”; however, no universal threshold has been established for these “adequate amounts” (FAO/WHO 2002). The frequently cited range of 10^6^–10^9^ CFU/g at consumption serves as a practical guideline, but the effective dose is strain‑specific and indication‑dependent (Hill et al. 2014). For athletic performance enhancement in healthy adults, daily doses ranging from 1 × 10^9^ to 1 × 10^11^ CFU have demonstrated significant effects, whereas for the prevention of ICU‐acquired infections, doses exceeding 22 × 10^9^ CFU/day administered for at least 14 days have been reported to be more effective (Al‐Aklabi et al. 2026; Zhang et al. 2026). In inflammatory bowel disease, doses lower than 10^10^ CFU/day administered for more than 8 weeks were associated with a greater reduction in the risk of relapse (W. Liu, Zhang, et al. 2025b). However, for irritable bowel syndrome symptom relief, no clear dose–response relationship has been consistently observed.

Regulatory agencies, including the European Food Safety Authority (EFSA) and the Brazilian Health Regulatory Agency (ANVISA), therefore require that the dose at the end of shelf life matches that proven effective in human intervention studies for each specific health claim. This dose–response variability reinforces the argument that maintaining high cell counts alone is insufficient to guarantee probiotic functionality.

To date, extensive evidence supports the beneficial effects of conventional probiotic genera (*Lactobacillus*, *Bifidobacterium*, and *Saccharomyces*) on immunity, gut health, and cancer prevention (Luo et al. 2020; Zhou et al. 2024; Torabi et al. 2025), while next‑generation probiotics such as *Akkermansia muciniphila*, *Faecalibacterium prausnitzii*, and *Clostridium butyricum* further expand the therapeutic scope by targeting metabolic and inflammatory conditions with greater specificity (Cao et al. 2019; Jan et al. 2024; Li et al. 2024). An overview of key species appears in Table 1.

**TABLE 1:** Probiotic microorganisms employed in foodstuffs (conventional and emerging).

Group	Genus	Species/strains
Yeasts	*Saccharomyces*	*S. cerevisiae*, *S. boulardii*
	*Debaryomyces*	*D. hansenii*
Filamentous fungi	*Aspergillus*	*A. niger*, *A. oryzae*
Lactic acid‐producing bacteria	*Lactobacillus*	*L. acidophilus*, *L. amylovorus*, *L. brevis, L. bulgaricus*, *L. cellobiosus*, *L. crispatus*, *L. curvatus*, *L. fermentum*, *L. gallinarum*, *L. helveticus*, *L. johnsonii*, *L. lactis*, *L. gasseri, L. sporogenes*, *L. pentosus*, *L. sanfranciscensis*, *L. farciminis*, *L. vaginalis*, *L. delbruecki*i subsp. *bulgaricus*
	*Lacticaseibacillus*	*L. casei*, *L. paracasei*, *L. rhamnosus* (GG)
		*L. plantarum*
	*Limosilactobacillus*	*L. reuteri*, *L. fermentum*
	*Pediococcus*	*P. acidilactici*, *P. pentosaceus*, *P. cerevisiae*
	*Streptococcus*	*S. faecalis*, *S. cremoris*, *S. lactis*, *S. intermedius*, *S. thermophilus*
	*Lactococcus*	*L. lactis* subsp. *lactis*, *L. lactis* subsp. *cremoris*
	*Leuconostoc*	*L. turleri*, *L. mesenteroides*
	*Enterococcus*	*E. durans*, *E. faecium*, *E. gallinarum*, *E. faecalis*
	*Ligilactobacillus*	*L. salivarius*
Bifidobacteria	*Bifidobacterium*	*B. adolescentis*, *B. animalis* subsp. *lactis*, *B. bifidum*, *B. breve*, *B. essensis*, *B. longum* subsp. *infantis*, *B. longum*, *B. laterosporum*, *B. thermophilum*
Propionic acid bacteria	*Propionibacterium*	*P. turleri*, *P. shermanii* subsp. *freudenreichii*
Bacteroides	*Bacteroides*	*B. capillus* ^a^, *B. suis* ^a^, *B. ruminicola* ^a^, *B. amylophilus* ^a^, *B. fragilis* ^a^, *B. uniformis* ^a^
Enterobacteria	*Escherichia*	*E. coli* (including *E. coli* Nissle 1917)
Spore‐forming bacteria	*Bacillus*	*B. alcalophilus*, *B. cereus*, *B. clausii*, *B. coagulans*, *B. subtilis*, *B. pumilus*, *B. lentus*, *B. licheniformis*
Clostridia	*Clostridium*	*C. butyricum*
Next‐generation probiotics (NGPs)	*Akkermansia*	*A. muciniphila*
	*Faecalibacterium*	*F. prausnitzii*
	*Eubacterium*	*E. hallii*
	*Christensenella*	*C. minuta*
	*Anaerobutyricum*	*A. soehngenii*
	*Prevotella*	*P. copri* ^a^
	*Oxalobacter*	*O. formigenes*
	*Roseburia*	*Roseburia* spp.
	*Parabacteroides*	*Parabacteroides* spp.^a^
Emerging LAB	*Weissella*	*Weissella* spp.

*Note*: Conventional probiotics (e.g., *Lactobacillus* and *Bifidobacterium*) are widely commercialized; next‐generation probiotics (NGPs) and emerging LAB are largely at the research stage.

^a^ Genera with emerging or debated probiotic status (e.g., *Bacteroides*, *Prevotella*, *Parabacteroides*), included based on current research interest rather than established probiotic status per FAO/WHO criteria.

Postbiotics and paraprobiotics—preparations of inanimate microbial cells and/or their components—have also gained attention for improved safety, stability, and suitability for vulnerable populations (Salminen et al. 2021; Vinderola et al. 2022). However, a substantial gap remains between the number of strains demonstrating in vitro or preclinical potential and those successfully commercialized (Table 2). A candidate strain must survive industrial production, tolerate fermentation, concentration, freezing, and drying, and often requires specific nutritional or atmospheric conditions that challenge cost‐effectiveness (Canon et al. 2020; Kwoji et al. 2022).

**TABLE 2:** Microorganisms currently commercialized as probiotics worldwide.

Group	Genus/species (examples)	Commercial applications	Product categories
Lactic acid bacteria (LAB)	*Lactobacillus delbrueckii* subsp. *bulgaricus*; *L. acidophilus*; *Lacticaseibacillus rhamnosus* (e.g., strain GG); *L. casei*; *Lactiplantibacillus plantarum*; *Limosilactobacillus reuteri*; *Streptococcus thermophilus*; *Pediococcus acidilactici*; *Leuconostoc mesenteroides*	Dairy products (yogurt, cheese, fermented milk), dietary supplements	Yogurt, cheese, fermented milk, capsules, sachets
Bifidobacteria	*Bifidobacterium bifidum*; *B. longum*; *B. breve*; *B. adolescentis*; *B. animalis* subsp. *lactis*	Infant formulas, functional dairy products, dietary supplements	Infant formula, yogurt, freeze‐dried powders, capsules
Enterococci (limited use, strain‐dependent)	*Enterococcus faecium* (e.g., strain SF68)	Veterinary probiotics, some dietary supplements	Animal feed, probiotic capsules
Spore‐forming bacteria	*Bacillus coagulans*; *B. subtilis*; *B. clausii*	Probiotic capsules, powders, functional foods	Capsules, cereal bars, baked goods, thermally processed foods
Yeasts	*Saccharomyces cerevisiae* var. *boulardii*	Capsules, sachets, prevention/treatment of diarrhea	Capsules, sachets, dairy drinks

*Note*: This table provides a general overview of commercialized probiotic microorganisms by taxonomic group. Daily doses, approved health claims and clinical evidence are highly strain‐specific and are not detailed here; representative examples are discussed in the main text and cited references.

After production, probiotics must maintain viability (typically 10^6^–10^9^ CFU/g) during storage, overcoming acidity, oxygen, and temperature fluctuations that heavily depend on the food matrix (Feng and Wang 2020; Treven et al. 2024). In the gastrointestinal (GI) tract, they face low pH, bile salts, and enzymes while competing with resident microbiota, often losing 3–6 log units before reaching the small intestine (Castro‐López et al. 2022; Bustos et al. 2024). This attrition explains why many promising in vitro strains fail to deliver benefits in practice.

Early efforts relied on empirical strain selection, but approaches have become more sophisticated and synergistic. Current strategies include (i) exploiting protective properties of food matrices (e.g., buffering in dairy, low water activity in chocolate); (ii) applying advanced microencapsulation with polysaccharides, proteins, or lipids to shield cells (Lopes et al. 2020; Rolim et al. 2020; Amiri et al. 2022); (iii) using nonthermal techniques such as high‐intensity ultrasound and high‐pressure homogenization to induce stress adaptation without major viability losses (Massoud et al. 2015; Gholamhosseinpour and Hashemi 2018; Mizuta et al. 2025); and (iv) employing synthetic biology and CRISPR‐based editing to engineer more robust, functionally targeted strains (Bravo and Landete 2017; Langa et al. 2022; Mugwanda et al. 2023; Z. Xie et al. 2026).

This review is guided by two central hypotheses. First, we propose that intrinsic matrix properties are key determinants of the observed variation in probiotic survival and functionality across food matrices. Second, we hypothesize that engineering stress tolerance pathways through approaches such as stress adaptation or genetic modification in combination with encapsulation technologies can enhance the survival and functionality of probiotics in processed foods.

This review critically evaluates these strategies and their synergistic potential, highlighting that successful probiotic incorporation depends on integrating strain selection, stress adaptation, matrix design, and technological innovations tailored to specific food systems and target populations.

## 2 Methodology for Literature Review

This review was conducted as a narrative review, given the broad and multidisciplinary scope of the topic, which encompasses strain selection, food matrices, encapsulation technologies, and genetic engineering strategies for probiotics. A systematic search was performed in the following electronic databases: PubMed, Scopus, Web of Science, and Google Scholar. The search covered the period from 2000 to March 2026, with priority given to articles published in the last 10 years (2016–2026) to ensure currency, although seminal older references were retained when historically relevant.

The following search terms and Boolean operators were used: (“probiotic” OR “lactic acid bacteria” OR “Bifidobacterium”) AND (“viability” OR “survival” OR “stability”) AND (“food matrix” OR “encapsulation” OR “microencapsulation” OR “spray drying” OR “freeze drying”) AND (“stress adaptation” OR “cross‐protection” OR “prebiotic” OR “synbiotic*”) AND (“genetic engineering” OR “CRISPR” OR “bioengineering”). Additional articles were identified by manual screening of reference lists of included studies and relevant reviews.

The inclusion criteria were as follows: (i) original research articles, review articles, and clinical studies published in peer‐reviewed journals; (ii) studies reporting quantitative data on probiotic viability or functionality in food systems; and (iii) articles written in English. The exclusion criteria were as follows: (i) conference abstracts; (ii) studies without original data or without clear methodological descriptions; and (iii) articles focused exclusively on clinical outcomes without technological relevance.

The literature screening was conducted independently by two authors (S.R.J.S. and R.C.R.). Titles and abstracts were first screened for relevance, followed by full‐text evaluation. Disagreements were resolved through discussion, and when consensus could not be reached, a third author (E.M.) was consulted.

Evidence levels were distinguished as follows: peer‐reviewed original research articles (in vitro studies, animal models, and human clinical trials) were considered primary evidence; review articles were used for contextual synthesis; industry reports and market data were cited only for economic context, not as evidence of scientific efficacy. Where commercial products or press releases are mentioned, these are explicitly identified as illustrative examples rather than controlled scientific evidence.

## 3 Limiting Factors for Probiotic Application in Foods

Although many microorganisms show probiotic potential (Table 1), several factors limit their incorporation into functional foods. Understanding these limitations is essential for designing effective strategies.

### 3.1 Safety and Compatibility With the Food Matrix

Qualifying a strain as a probiotic requires comprehensive safety and functional assessment beyond demonstrated health effects. The FAO/WHO (2002) criteria include molecular identification and in vitro evaluation of acid and bile tolerance, adhesion, antimicrobial activity, and bile salt hydrolase (Winger 2025). Expanded frameworks include genomic screening for antibiotic resistance genes, virulence factors, toxin production, hemolysis, and biogenic amines (Žuntar et al. 2020; Oba et al. 2022). Robust strains integrate GI resilience, adhesion, antimicrobial effects, and functional bioactivity (Kanak and Yılmaz 2025).

The incorporation of probiotics into food matrices remains challenging, as they may affect flavor, texture, and appearance, requiring compatibility assessments to preserve sensory quality (Barros et al. 2020; Grujović et al. 2025). Nevertheless, commercial applications demonstrate feasibility, including *Bifidobacterium lactis* in yogurts, *Lactobacillus reuteri* DSM 17938 in infant formulas, and multi‐strain blends in cereal bars. A concise overview of currently commercialized probiotic microorganisms, with their taxonomic classification and main applications, is presented in Table 2.

Probiotic metabolism influences sensory properties through organic acids, ethanol, diacetyl, exopolysaccharides (EPSs), and volatile compounds. Beyond cheese‐related aromas (e.g., volatile sulfur compounds from *Lactococcus lactis* and *Lactiplantibacillus plantarum*), probiotics produce diverse volatile profiles, including alcohols (such as 3‐methyl‐1‐butanol, contributing fruity notes), aldehydes (e.g., hexanal, associated with green/grassy aromas), ketones (e.g., 2‐heptanone, nutty/cheesy), and esters (e.g., ethyl acetate, fruity) (Bonnarme et al. 2004; Salmeron et al. 2009; Ramirez et al. 2025). In fermented milks, *Lacticaseibacillus paracasei* produces diacetyl (buttery aroma) and acetoin (creamy), while *Bifidobacterium animalis* generates acetic acid (vinegar‐like) and 2,3‐butanedione (caramel‐like) (Sun et al. 2024). Recent metabolomics has identified strain‐specific volatile markers enabling flavor prediction and quality control (Zhang et al. 2025). These findings underscore the need for strain‑ and matrix‑specific sensory evaluation.

### 3.2 From Processing to GI Transit: The Viability–Functionality Gap

The second limiting factor is the ability of probiotics to maintain viability from processing through to consumption. Processing conditions are, by far, the primary cause of probiotic inactivation, as cells are exposed to successive and cumulative stresses throughout the manufacturing process (Kiepś and Dembczyński 2022; A. Wang and Zhong 2024). For example, high‐temperature short‐time pasteurization (72°C, 15 s) reduces *Lacticaseibacillus casei* viability by approximately 1.5–2.0 log CFU/mL in milk, while ultrahigh temperature processing (135°C, 2–4 s) causes nearly complete inactivation (4–5 log reduction) (Champagne et al. 2018). In spray drying, outlet air temperatures of 70–80°C result in 1–3 log reductions in the viability of most lactobacilli (A. Wang and Zhong 2024), whereas freeze drying, despite being a milder process, results in 0.5–1.5 log reductions in the viability of *Bifidobacterium* (Saarela et al. 2006).

Furthermore, matrix‐specific factors also affect survival, as observed in fermented foods, where parameters such as temperature, moisture, pH, and osmotic pressure support microbial growth (Cabello‐Olmo et al. 2020; Y. Liu, Liu, et al. 2025a). However, in matrices with limited metabolic interaction, significant viability loss may occur, requiring high initial inoculum levels (10^6^–10^8^ CFU/g) to compensate for processing‐related mortality (Treven et al. 2024). In fruit juices with low pH (3.0–4.0), for example, *Lactobacillus rhamnosus* GG exhibits a reduction of 2–4 log CFU within 14 days (Maia et al. 2023).

The inactivation of probiotics after processing is mainly associated with oxidative stress and reactive oxygen species (ROS), which damage cellular structures (Mbye et al. 2020; Castro‐López et al. 2022). In fermented milks with oxygen levels as low as 5–10 ppm, the viability of *B. animalis* subsp. *lactis* is reduced by 1–2 log over 28 days under refrigeration, whereas oxygen‑impermeable packaging preserves counts (Feng and Wang 2020).

It is worth noting that processing, matrix composition, and storage may impair probiotic functionality even when viable counts remain adequate (Champagne et al. 2018). It has been documented that after processing, *Bifidobacterium* strains and *L. casei* may lose bile salt hydrolase activity and adhesion capacity, even when viable counts remain above 10^6^ CFU/g (Saarela et al. 2006; J. Wang et al. 2009; Aktas et al. 2022). It has been shown that the acid and bile tolerance of freeze‐dried *B. animalis* subsp. *lactis* Bb‐12 cells stored in low‐fat milk declines during storage at refrigeration temperature, but high culturability of the cells is maintained in the food matrix (Saarela et al. 2006). Moreover, the choice of cryoprotectant (sucrose vs. skim milk) affected survival in juice and milk matrices differently, indicating that both processing and matrix composition affect functional properties. J. Wang et al. (2009) showed that *L. casei* Zhang exhibited high viability (> 10^8^ CFU/g) during storage in soymilk as well as bovine milk; however, the variation in physicochemical properties of these matrices (e.g., buffering capacity) affected fermentation characteristics and pH stability. Together, these results suggest that viability maintenance is not required for retaining functional properties and that processing conditions and matrix composition have different effects on probiotic functionality.

In contrast, some studies have reported that the functionality of probiotics after processing can be positively influenced by the food matrix (Vinderola et al. 2011; Dias et al. 2023). This is the case for *L. rhamnosus* GG, which exhibits greater adhesion to intestinal cells when delivered in a dairy‐based matrix than in other matrices (Guerin et al. 2017; Hellebois et al. 2024). Similarly, Pavunc et al. (2023) reported that *L. casei* 431 exhibited greater inhibitory activity against foodborne pathogens and increased cholesterol assimilation (16.4%) when delivered in a food matrix compared to a lyophilized culture, whereas bile salt hydrolase activity was only minimally affected. These results highlight that the matrix effects on probiotic functionality are strain‐ and matrix‐specific and may either improve or reduce the functional properties, depending on the matrix composition and strain characteristics.

Overall, the co‐ingested food matrix has been reported to play a modulatory role in probiotic survival, with solid and semisolid foods providing buffering capacity and physical protection during gastric transit, thereby enhancing survival relative to liquid carriers or fasting conditions (Treven et al. 2024).

Beyond the factors previously described, GI transit may impose additional stresses on injured cells, thereby further compromising probiotic functionality, including immunomodulatory effects, pathogen inhibition, and enzymatic activity. During GI transit, probiotic microorganisms are exposed to sequential pH gradients, digestive enzymes such as pepsin and pancreatin, and varying concentrations of bile salts (Bustos et al. 2024; Dimitrellou et al. 2025). Additional stressors, including oxygen tension, nutrient limitation, high osmolarity, ROS, and peristalsis‐induced mechanical forces, further compromise cell viability (Castro‐López et al. 2022).

Compared with other probiotic strains, spore‐forming bacteria such as *Bacillus coagulans* and *Bacillus clausii* possess a distinct advantage, as their dormant spores are highly resistant to low pH, elevated temperatures, and mechanical stress and germinate only upon reaching the small intestine (Ke et al. 2026). Similarly, eukaryotic probiotics generally exhibit greater resistance to adverse GI conditions because of their robust cell wall structure. This has been well‐documented for *Saccharomyces boulardii* (taxonomically classified as *Saccharomyces cerevisiae* var. *boulardii*), which displays high intrinsic resistance to gastric acidity and bile salts (J. Wang, Wang, et al. 2025b).

In the GI tract, the resident microbiota may represent an additional stress factor, as it exerts colonization resistance through competition for niches and nutrients and the production of antimicrobial metabolites, thereby limiting the persistence of exogenous probiotics (Caballero‐Flores, Pickard and Núñez. 2022; Horrocks et al. 2023).

Collectively, these physicochemical, physiological, and ecological barriers preclude the existence of a single probiotic strain capable of meeting all performance requirements. These findings support the notion that viability alone is insufficient to guarantee probiotic functionality, which encompasses resistance to GI conditions, adhesion to the intestinal mucosa, immunomodulatory activity, pathogen inhibition, and enzymatic activity. This important aspect is often overlooked in studies that report only viable cell counts.

### 3.3 Regulatory Framework and Dosage Requirements for Probiotics

Beyond scientific criteria, regulatory frameworks define requirements for probiotic health claims and viable counts. The EFSA requires strain identification by whole‐genome sequencing, safety assessment (absence of antibiotic resistance genes, virulence factors, and toxins), and at least one human intervention study demonstrating health benefits (EFSA 2022). In the United States, the Food and Drug Administration (FDA) applies the generally recognized as safe (GRAS) framework, requiring safety evidence based on scientific data or a history of safe use (FDA 2022).

Although the FAO/WHO (2002) suggests 10^6^–10^9^ CFU/g at consumption, regulatory agencies do not universally mandate fixed thresholds. Instead, they require that the administered dose match that is effective in clinical studies, reflecting a strain‐specific dose–response relationship (Hill et al. 2014).

In Brazil, ANVISA requires the declaration of viable counts at the end of shelf life and scientific substantiation of health claims, without establishing minimum thresholds (ANVISA 2023). This variability highlights the need for harmonized international guidelines to support global commercialization and consumer protection.

## 4 Strategies to Improve the Viability of Probiotics in Food Matrices

Improving probiotic viability in foods can be achieved through strategies ranging from selecting naturally resistant strains to applying protective technologies such as encapsulation.

### 4.1 Strain Selection and Intrinsic Stress Tolerance

Strain selection relies on screening methods that simulate harsh conditions during processing and storage, including heat, low pH, and high levels of lactic acid, ethanol, and NaCl (Ramírez‐Cota et al. 2020; Cizeikiene and Jagelaviciute 2021). Strains with higher intrinsic resistance are preferred, as they ensure stability and survival without requiring complex protective technologies.

Recent studies on fermented buffalo milk identified highly tolerant strains: *L. plantarum* Y1 showed strong acid (> 5.0 log CFU/mL at pH 2.0) and bile tolerance (6.5 log CFU/mL), while *L. brevis* Cc3 combined bile resistance (6.0 log CFU/mL) with antioxidant activity (52% DPPH scavenging). Nonconventional isolates such as *Bacillus dendritiformis* Y9 also demonstrated notable bile tolerance (5.4–5.5 log CFU/mL), expanding potential probiotic candidates (Saleem et al. 2025). However, as previously mentioned, high viability does not guarantee probiotic functionality during administration.

Spores of *B. coagulans* MTCC 5856 exhibit high thermal resistance (*D* value 35.71 at 90°C) and maintain viability during heat processing, supporting their use in thermally processed foods (Majeed et al. 2020). In addition, tolerance to salt and acidity is critical for lactic acid bacteria (LAB), as fermentation increases osmotic stress; strains resistant to NaCl better withstand salinity variations (Liao et al. 2023; Ndiaye et al. 2024). For example, cheese‐related strains must tolerate 4%–6% NaCl and broad pH ranges (Tidona et al. 2022).

### 4.2 Stress Adaptation and Cross‐Protection Mechanisms

Probiotic robustness can be enhanced by exposure to sublethal stresses through a phenomenon known as cross‐protection: exposure to one mild stress triggers global stress responses that increase tolerance to other, more severe stresses (Bustos et al. 2024). This is mediated by the upregulation of stress‐related genes, chaperones (GroEL and DnaK), and adjustments in membrane fatty acid composition. For example, Huo et al. (2025) demonstrated that brief exposure to oxidative, acid, osmotic, or bile stress increased the heat resistance of *Ligilactobacillus salivarius* SS1 at 70°C by 1.3‐ to 1.6‐fold (85.7%–98.9%), supporting preadaptation strategies for industrial processes. For context, nonadapted cells typically show only 45%–55% survival under the same heat challenge, meaning that acid preadaptation nearly doubles viable cell recovery (Huo et al. 2025).

Similarly, *L. plantarum* preadapted to pH 5.0 maintained viability for 15 days in synthetic wine, unlike nonadapted cells (Succi et al. 2017). *L. lactis* exposed to osmotic (8% NaCl), acidic (pH 4.5), or oxidative (1 mM H_2_O_2_) stress showed improved resistance during drying and storage (Cnossen et al. 2020). Quantitatively, oxidative preadaptation reduced spray drying losses to only 0.01 log (vs. 0.38 log in controls), and after 45 days of storage, adapted cells showed 112.5% higher viability (Cnossen et al. 2020). Sublethal high‐pressure homogenization (50 MPa) also enhanced the survival of *L. paracasei* A13 in cheese and under simulated gastric conditions (Burns et al. 2008), an effect mediated by upregulation of fatty acid biosynthesis genes (*accA*, *fabD*, and *fabZ*) that enhance membrane integrity (Siroli et al. 2020). These effects are linked to the activation of stress‐response systems, including chaperones (GroEL and DnaK), antioxidant enzymes, and compatible solute transporters (Mbye et al. 2020; Castro‐López et al. 2022).

Metabolomic analysis of *L. casei* AP under thermal stress revealed strain‐specific adaptations, such as increased production of nucleotides, peptides, and membrane‐stabilizing compounds, contributing to enhanced resilience (Rakhmatulloh et al. 2026). In *L. plantarum* KLDS 1.0628, heat adaptation (45°C for 1 h) produced a 31.38‑fold increase in thermal tolerance (Ma et al. 2021), highlighting that the degree of protection varies widely by strain. These findings support the use of stress adaptation to improve probiotic stability during industrial processing.

## 5 Application of Probiotics in Food Systems

The incorporation of probiotics into foods depends on selecting suitable matrices and applying protective technologies. This section addresses these aspects: key probiotic carriers and strategies to enhance survival.

Different food matrices provide varying probiotic protection based on physicochemical properties. Grujović et al. (2025) highlight that understanding strain–matrix interactions is essential for developing stable, effective products, emphasizing dairy, meat, cereals, and plant‐based beverages. Nikitina and Khrundin (2026) report that appropriate carriers enhance microbial stability, modulate proteolysis and lipolysis, and improve antioxidant and antimicrobial properties without compromising sensory quality, particularly in plant‐based matrices for lactose‐intolerant and vegetarian populations. A quantitative summary of probiotic survival across different food matrices, including representative outcomes and key references, is presented in Table 3.

**TABLE 3:** Summary of probiotic survival across food matrices: Mechanisms, outcomes, and key references.

Matrix	Probiotic strain/technology	Condition	Survival outcome	Key references
Dairy (milk chocolate)	*L. acidophilus* LDMB‐01	90 days at 4°C	> 6 log CFU/g	Islam et al. (2022)
Fruit juice (acerola)	*B. animalis* BB‐12 (encapsulated vs. free)	30 days at 4°C	8 log vs. 5.9 log CFU/200 mL	de Paula Antunes et al. (2025)
Fruit juice (apple)	*L. rhamnosus* GG (whey + starch)	5 weeks at 4°C–25°C	Viability maintained	Ying et al. (2013)
Fruit juice (pineapple)	*L. paracasei* Shirota (alginate beads)	Refrigerated storage	Good GI tolerance	Rani et al. (2025)
Cereal (protein bar)	*L. acidophilus* LA‐2 (κ‐carrageenan + inulin)	Refrigerated storage	High viability maintained	Chen and Mustapha (2012)
Cereal (baked)	*B. coagulans* BC4 (bean‐rice)	Baked at 180°C	9.26 ± 0.28 log CFU/g	Bento et al. (2026)
Cereal (baked)	*B. coagulans* BC4 (oat)	Baked at 180°C	8.62 ± 0.14 log CFU/g	Bento et al. (2026)
Cereal (noodles)	*B. coagulans* GBI‐30	After boiling (7 min)	5.75 log CFU/g	J. Wang, Wu, et al. (2025d)
Cereal (noodles)	*L. rhamnosus* GG	After boiling (7 min)	6.88 log CFU/g	J. Wang, Wu, et al. (2025d))
Meat (sausage)	*L. plantarum* + dextrose + starch	12 days at 4°C	Extended shelf life	Pipaliya and Yadav (2025)
Meat (salami)	*L. rhamnosus* GG (encapsulated)	After simulated digestion	2 log loss (vs. 3 log free)	Biasuz et al. (2025)
Chocolate (dark)	*S. boulardii* (encapsulated vs. free)	120 days storage	2.51 log higher	Kanak and Yılmaz (2026)
Chocolate (dark)	*S. boulardii* (encapsulated vs. free)	Simulated gastric (pH 3, 2 h)	3.99 log vs. 6.42 log loss	Kanak and Yılmaz (2026)
Chocolate (synbiotic)	*L. acidophilus* La‐14 + corn/honey	> 125 days	Viability maintained	Singh and Gaur (2025)
Chocolate (NGP)	*A. muciniphila* (54.5% cocoa)	28 days aerobic	> 10^6^ CFU/g	Vedor et al. (2023)
Chocolate (synbiotic)	SLAB51 multi‐strain	6 months at 25°C	42.5% survival (1.7 × 10^10^ CFU/30 g)	Suo et al. (2026)

*Note*: Data shown are representative examples; survival varies by strain, matrix, and processing conditions.

Abbreviation: NGP, next‐generation probiotic.

It is important to emphasize that the survival data presented in Table 3 and throughout this section represent strain‐ and matrix‐specific outcomes reported in the cited studies. These findings should not be interpreted as universally applicable to all probiotic strains within a given genus or species. Probiotic performance varies considerably depending on strain‐specific genetic traits, metabolic activity, and physiological state at the time of processing. Therefore, the strategies and outcomes discussed here should be viewed as illustrative examples that provide general guidance rather than predictive rules.

### 5.1 Probiotic Survival Across Different Food Matrices

Several studies directly compare the same probiotic strain across different matrices, providing explicit guidance for matrix selection. For example, *L. rhamnosus* GG showed markedly different survival depending on the carrier: casein provided the highest protection (8.50 log CFU/g after GI digestion), starch conferred moderate protection (5.81 log CFU/g), whereas soybean oil resulted in bead dissolution and undetectable viability (Udo et al. 2025). This demonstrates that protein‐based matrices, such as dairy products, offer superior protection for this strain compared to lipids or carbohydrates.

Dairy products are widely considered the “gold standard” for probiotic delivery due to their high buffering capacity (pH 4.0–4.5), largely provided by caseins and phosphates, which mitigate pH declines, as well as their casein network, which restricts the diffusion of acids and bile salts, and their fat globules, which create hydrophobic microenvironments that reduce cellular exposure to acidic conditions (see Table 3).

However, some nondairy matrices can approximate this performance under specific conditions. For example, coconut water fermented with *Bifidobacterium* strains can reach viable counts exceeding 12 log CFU/mL after fermentation and retain counts above 6 log CFU/mL following 42 days of storage at 4°C (Jahajeeah and Choo 2024; Santos et al. 2025). Similarly, soymilk fermented with *L. rhamnosus* achieved viability of 10.47 log CFU/mL (free cells) and 12.98 log CFU/mL (synbiotic immobilized cells), with superior GI tract survival compared to free cells (Gad et al. 2024).

In plant‐based matrices, spores of *B. coagulans* maintain counts above 8 log CFU/g in cereal products after cooking and long‐term storage due to their inherent thermoresistance (Bento et al. 2026; J. Wang, Wu, et al. 2025d), whereas viability declines more rapidly in fruit beverages. This can be attributed to the fact that cereal matrices protect probiotics primarily through their low water activity (aw 0.3–0.7), which limits metabolic activity, and through the presence of prebiotic fibers that provide physical stabilization (Table 3).

In a similar manner, probiotic protection in chocolate is based on its low water activity (aw < 0.5), which limits metabolic activity; however, this class of products also benefits from additional protective factors, as the high fat content provides a hydrophobic barrier against gastric acid, and cocoa polyphenols contribute to antioxidant activity (Table 3).

In contrast, fruit and vegetable beverages provide minimal intrinsic protection due to their low pH (2.5–4.0) and lack of buffering components (Table 3). For example, in orange juice (pH 2.5–4.0, low solids, and no fat), populations of *L. paracasei* Shirota and *L. rhamnosus* GG decreased to 3.80 log CFU/mL during simulated GI transit, whereas in mango juice, *L. plantarum* exhibited a reduction of 3.43 log CFU/mL over 28 days of storage (Nazir et al. 2024; Vitsou‐Anastasiou et al. 2025). However, incorporating prebiotics such as orange fiber and mango juice can significantly improve *L. casei* viability above 7 log CFU/mL during 45 days of refrigerated storage (Roufegarinejad et al. 2022). The protective effect of mango juice is probably due to the presence of soluble fiber, which offers physical protection and a fermentable substrate, while orange fiber contributes to pH buffering and diminishes exposure to oxygen due to its structural matrix.

In meat products, probiotic incorporation remains challenging because these foods are typically cooked at high temperatures (e.g., internal temperatures of 70°C–80°C) to ensure microbiological safety, whereas such conditions can inactivate most vegetative probiotic cells. Studies have shown that *B. coagulans* and *B. subtilis* spores supplemented in cooked sausages retained high viability after boiling (8.48–9.11 log CFU/10 kg), microwaving (7.92–8.95 log CFU/10 kg), and deep‐fat frying (7.63–8.76 log CFU/10 kg) (Jafari, Mortazavian, and Hosseini 2017; Payne 2024). However, this high survival is primarily attributable to the inherent thermoresistance of the spore form, rather than to a protective effect of the meat matrix itself. In fact, probiotic vegetative cells such as *Lactobacillus* and *Bifidobacterium*, which are not spore‐forming, usually show considerable decreases under similar cooking conditions (Jafari, Mortazavian, and Hosseini 2017; Champagne et al. 2018; Kiepś and Dembczyński 2022). Jafari, Mortazavian, and Hosseini (2017) reported a 3–4 log reduction in spore counts after cooking, indicating that even spore‐forming strains are subject to significant losses. Therefore, the use of spore‐forming strains in addition to an optimized formulation of the meat matrix appears to be the most promising approach for probiotic delivery in cooked meat products.

In fermented meat products, which typically exhibit a pH of 5.5–6.0 and a water activity of 0.90–0.95, the fat–protein matrix can enhance probiotic survival (Table 3). Fermented sausages offer an environment conducive to the growth and survival of probiotics, and viable counts can reach 10^7^–10^8^ CFU/g without heat treatment (Mati et al. 2015; Mani‐López et al. 2024). On the other hand, fresh and cooked meat products are subjected to heating and the use of additives that can compromise probiotic viability, making probiotic incorporation more difficult in these matrices (Pasqualin Cavalheiro et al. 2015). A significant advantage of fermented meats as a vehicle for probiotic delivery is the absence of heat treatment, which typically causes considerable inactivation of vegetative cells in cooking steps. Fat globules create hydrophobic domains that reduce exposure to gastric acid and bile salts, while protective compounds such as nitrites, spice‐derived phenolics, and peptides generated during fermentation further contribute to cell stability. Nevertheless, oxidative stress remains a significant challenge during storage.

The performance gap between dairy and challenging nondairy matrices can be closed through (i) encapsulation—alginate, whey protein, or pectin‐based microcapsules provide physical protection and pH buffering, as demonstrated in orange juice (encapsulated probiotics maintained 6.80–7.00 log CFU/mL vs. 3.80 log for free cells during GI simulation) (Vitsou‐Anastasiou et al. 2025); (ii) prebiotic inclusion—inulin, FOS, or orange fiber act as cryoprotectants during storage and fermentable substrates in the gut (Jahajeeah and Choo 2024); and (iii) fat supplementation—babassu coconut milk (rich in lauric and myristic acids) combined with cashew apple maintained *L. casei* viability above 7.00 log CFU/mL during 42 days of refrigerated storage, demonstrating that high‐fat plant matrices can mimic dairy protection.

### 5.2 Technological Strategies to Enhance Probiotic Survival

#### 5.2.1 Prebiotics as Synergistic Protectors

To maintain high probiotic populations and support intestinal functionality, prebiotics are frequently incorporated as growth promoters (Terpou et al. 2019; Rosa et al. 2021; S. You et al. 2022). Common prebiotics include FOS, GOS, xylooligosaccharides (XOS), inulin, resistant starch, pectin, and β‐glucans (Senés‐Guerrero et al. 2020).

Recent studies highlight their role in enhancing probiotic viability and functionality, particularly in plant‐based and dairy systems (Sionek and Szydłowska 2025; Parhi et al. 2024). Synbiotic combinations promote synergistic effects, improving survival, colonization, and health benefits such as digestion, immunity, and inflammation control (Jena and Choudhury 2025).

Prebiotics act through multiple mechanisms: as fermentable substrates that stimulate beneficial microbiota, as cryoprotectants during freezing and drying, and as structural components that create protective microenvironments against oxygen and acidity. In dairy matrices, inulin and FOS enhance probiotic viability and functionality, improving survival during storage and simulated digestion while promoting short‐chain fatty acid production (Rolim et al. 2020; D'Almeida et al. 2024; Parhi et al. 2024). In frozen dairy systems, inulin improves the survival of *Lactobacillus* and *Bifidobacterium* during storage and simulated digestion by acting as both a fermentable substrate and cryoprotectant (Rolim et al. 2020; D'Almeida et al. 2024). In cheese matrices, inulin and FOS promote higher populations of LAB and *Bifidobacterium* throughout ripening and stimulate short‐chain fatty acid production in colonic models, reinforcing their role as stable functional delivery systems (D'Almeida et al. 2024).

It is important to distinguish between two distinct roles of prebiotics in probiotic delivery. First, during storage in low‐water‐activity matrices (e.g., protein bars, aw < 0.3), prebiotics such as inulin and FOS act primarily as cryoprotectants, stabilizing cell membranes during freezing and drying without supporting probiotic proliferation (Bodzen et al. 2021). Second, upon consumption, prebiotics serve as fermentable substrates in the GI tract, stimulating short‐chain fatty acid production and enhancing probiotic survival during GI transit (Raval and Archana 2024; Pichamon et al. 2025).

Three classic studies illustrate these effects: Akın et al. (2007) reported that 2% inulin in probiotic ice cream improved the viability of *L. acidophilus* LA‐14 and *B. lactis* BL‐01, extending shelf life; Padilha et al. (2016) observed enhanced growth of *Lactobacillus* in petit‑suisse cheese enriched with inulin and FOS; and Langa et al. (2019) showed that while FOS did not increase the *L. paracasei* population in semihard cheese after 28 days, it improved microbial viability in a colonic model, indicating that prebiotic effects may manifest primarily during GI transit rather than during product storage. More recently, Y. Liu, Liu, et al. (2025a) demonstrated that complex prebiotics not only improved the physicochemical and sensory properties of fermented milk but also enhanced ACE inhibitory activity (up to 73.74%), α‐amylase inhibition (27.69%), and antimicrobial effects against foodborne pathogens, reinforcing that prebiotics contribute to both probiotic survival and functional performance. Plant‑derived prebiotics, such as hawthorn extract, further enhance growth and antioxidant activity in fermented milk (Utebaeva et al. 2025).

Beyond prebiotics of plant origin, such as inulin and FOS, plant‐derived extracts have also demonstrated protective effects, as reported by Vodnar and Socaciu (2014), who found that co‐encapsulation with selenium‐enriched green tea extract (2 g/100 mL) significantly enhanced (*p* < 0.05) the survival of *L. casei* and *L. plantarum* during refrigerated storage and simulated GI conditions. Similarly, Chaikham (2015) showed that cashew flower extract (0.05% w/v) significantly improved the viability of *L. casei* 01, *L. acidophilus* LA5, and *B. lactis* Bb‐12 in fruit juices and yogurt over 30 days at 4°C. Gaudreau et al. (2016) further demonstrated that co‐encapsulation with green tea extract enhanced *L. helveticus* survival under simulated GI conditions. The protective effects of these plant‐derived extracts can be attributed to multiple mechanisms: (i) their polyphenolic compounds provide antioxidant activity that reduces oxidative stress during processing and storage (Holkem et al. 2023); (ii) they may serve as fermentable substrates that stimulate probiotic growth and metabolic activity (Tleuova et al. 2025); and (iii) their antimicrobial properties can inhibit spoilage organisms, creating a more favorable environment for probiotic survival. These combined effects explain why plant‐derived prebiotics and extracts can enhance probiotic viability and functionality in food matrices.

Prebiotics can also influence the metabolic activity of probiotics, as demonstrated by Garavand et al. (2022), who reported that yogurts supplemented with spirulina and galactofructose enhanced γ‐aminobutyric acid (GABA) production, as well as the antioxidant and antihypertensive activities of *L. paracasei*. In addition, nondairy oligosaccharides have been shown to modulate the infant gut microbiota and support probiotic viability (Roberfroid et al. 2010; Robinson 2019).

Beyond digestive health, emerging evidence supports the role of probiotics, prebiotics, and synbiotics in mental health via the gut–brain axis. Chalotra et al. (2026) discussed therapeutic approaches for metabolic syndrome through gut microbiota modulation, emphasizing mechanistic roles across gut–brain, gut–liver, gut–immune, and gut–endocrine pathways. Li et al. (2024) further highlighted that probiotics and their derivatives (prebiotics, synbiotics, postbiotics, paraprobiotics) regulate gut microbiota balance and promote host health.

The importance of preserving both viability and functionality during processing is reinforced by Sardar et al. (2025), who demonstrated that lyophilized strains of *Bacillus*, *Lactobacillus*, and *Staphylococcus* stored without cryoprotectants or at higher temperatures exhibited significant viability loss and functional decline, including reduced adhesion potential, antimicrobial activity, and metabolic stability. Conversely, strains preserved with optimized cryoprotectant formulations (which often include prebiotic compounds such as inulin, trehalose, or FOS) maintained both viability and functionality over 12 months, highlighting that preservation conditions must be tailored to preserve functional attributes, not merely cell counts (Sardar et al. 2025).

Overall, prebiotics enhance probiotic survival, functionality, and health‐promoting effects. Nevertheless, further research is needed to optimize strain‐specific synbiotic combinations, validate their efficacy through well‐designed clinical studies, and identify novel plant‐derived prebiotics for targeted applications. As discussed in Section 3.2, preserving functionality alongside viability is critical; prebiotics can contribute to both objectives.

#### 5.2.2 Probiotic Encapsulation: Principles, Mechanisms, and Technology Selection

The choice of encapsulation strategy is inherently linked to the specific food matrix and its physicochemical properties, making the following techniques directly applicable to the food systems covered in this review. Encapsulation protects probiotics through four primary mechanisms: (i) a physical barrier—the encapsulating matrix (polysaccharides, proteins, lipids) shields cells from gastric acid, bile salts, and digestive enzymes; (ii) pH‐responsive release—materials such as alginate swell or dissolve at intestinal pH (6.5–7.5), enabling targeted delivery to the colon; (iii) mechanical protection—immobilization reduces cell damage during spray drying, freeze drying, and high‐pressure processing; and (iv) oxygen control—hydrophobic materials (e.g., lipids, chitosan) limit oxygen diffusion, protecting anaerobic bifidobacteria.

##### 5.2.2.1 Technology Selection for Specific Applications

The choice of encapsulation method depends on the target food matrix and processing conditions. In general, spray drying is suitable for producing probiotic powders for dry food applications (cereals, chocolate) but requires high inlet temperatures (160°C–200°C) that damage nonspore‐forming cells; viability retention ranges from 60% to 90% depending on protectant use ((A. Wang and Zhong 2024; de Deus et al. 2024). In terms of cost and scalability, spray drying is the most scalable technique (continuous operation, high throughput), making it preferred for large‐scale production of probiotic powders and dairy ingredients. On the other hand, freeze drying preserves viability (70%–95%) but is expensive and batch‐oriented, making it suitable for high‐value products (pharmaceuticals, premium supplements) (Amiri et al. 2022; Jin and Wang 2026).

Extrusion (ionic gelation) and electrospraying are mild encapsulation techniques that can be implemented at relatively low cost. While electrospraying produces highly uniform nano‐ to microsized particles, extrusion generates larger capsules that are particularly suitable for dairy and meat products (Amiri et al. 2022). However, both technologies remain largely confined to pilot‐scale production, and the large capsule size associated with extrusion (∼1 mm) can create texture‐related challenges in many foods (Rodrigues et al. 2020; A. Xie et al. 2023; Laina et al. 2025; D'Amico et al. 2025).

Emulsification is readily scalable and enables the production of smaller particles (10–100 µm) with improved oxygen barrier properties, making it suitable for lipid‐rich matrices and pharmaceutical formulations (Barajas‐Álvarez et al. 2021; Mavaddat and Zandkarimi 2026). Coacervation offers high encapsulation efficiency and controlled release properties, but its complexity and high cost generally restrict its application to specialized intestinal delivery systems (Cakmak et al. 2023; Bu et al. 2025). Multilayer nanocoating, in turn, provides superior protection and tunable release through layer‐by‐layer assembly. Nevertheless, its multistep fabrication process limits scalability, and its application remains largely confined to laboratory‐scale systems (Ji et al. 2025; Jin and Wang 2026).

Table 4 provides a quantitative comparison of these techniques, including typical particle sizes, viability retention ranges, scalability, and common applications. Recent advances have expanded the encapsulation toolkit: nanocoating systems enable layer‑by‑layer assembly for enhanced protection (Ji et al. 2025); chitosan‑based biopolymers offer site‑specific release in the GI tract (Hussain et al. 2025); and polysaccharide‑based microcapsules provide tunable degradation profiles (Yang et al. 2025). Industrial implementation faces challenges, including scale‑up costs, batch consistency, and preservation of sensory properties (Nikitina and Khrundin 2026; Hussain et al. 2025). For therapeutic applications, encapsulation enables controlled intestinal release and modulation of dysbiosis (Y. You et al. 2025).

**TABLE 4:** Quantitative comparison of encapsulation techniques for probiotics: Principles, materials, and applications.

Technique	Principle	Typical particle size	Viability retention	Scalability	Common materials	Advantages	Disadvantages	Protection mechanism	Food applications	Key references
Spray drying	Atomization and hot air drying	10–100 µm	60%–90% (with protectants)	High (continuous)	Maltodextrin, gum arabic, WPC, inulin	Low cost, continuous, scalable	Thermal and osmotic stress	Formation of vitreous matrix that immobilizes cells	Fermented milk powders, fruit juice powders	A. Wang and Zhong (2024), Kanimozhi and Sukumar (2023)
Freeze drying	Freezing and sublimation	10–1000 µm	70%–95%	Low (batch)	Sucrose, trehalose, skim milk, peptones	High viability preservation	High cost, batch process, slow	Cryoprotection by sugars that stabilize membranes	Starter cultures, probiotic powders	Amiri et al. (2022), Kanimozhi and Sukumar (2023)
Extrusion	Droplet formation in gelling solution	1–3 mm	85%–95%	Medium	Alginate, carrageenan, pectin, chitosan	Mild conditions, low cost, large capsules	Scale‐up difficulty, large capsule size	Ionic gelation forms hydrogel that protects at acidic pH	Cheeses, ice creams, meat products	Rodrigues et al. (2020), A. Xie et al. (2023)
Emulsification	Dispersion of aqueous phase in oil	10–100 µm	70%–90%	High	Alginate, chitosan, κ‐carrageenan, lipid‐based materials	Scale‐up potential, controllable size, oxygen barrier	Oil residue, solvent use	Water‐in‐oil emulsion with internal gelation; hydrophobic barrier	Meat products, lipid matrices, pharmaceutical formulations	Barajas‐Álvarez et al. (2021), Mavaddat and Zandkarimi (2026)
Electrospraying	Electric field for droplet formation	100 nm–100 µm	80%–95%	Low (lab scale)	Alginate, pectin, chitosan, gelatin	Uniform size (nano/micro), mild conditions, no heat	Low throughput, high investment	Room‐temperature encapsulation preserves cell integrity	High‐value functional yogurts	Laina et al. (2025), D'Amico et al. (2025)
Coacervation	Polymer phase separation	50–500 µm	70%–85%	Low	Gelatin‐gum arabic, chitosan‐alginate	High efficiency, controlled release	Complexity, cost	Polyelectrolyte complex formation reinforces capsule wall	Intestinal release matrices	Cakmak et al. (2023)
Multilayer nanocoating	Layer‐by‐layer assembly	10–1000 nm	80%–95%	Low (lab scale)	Chitosan, alginate, polyelectrolytes	Superior protection, tunable release	Multistep process, complexity	Electrostatic interactions form robust shells	High‐value functional foods	Ji et al. (2025)

A critical but often overlooked aspect is that encapsulation must preserve not only viable counts but also functional properties, as sublethal injury during the encapsulation process can compromise functionality even when viability remains high.

##### 5.2.2.2 Practical Considerations for Probiotic Encapsulation: Regulatory, Sensory, and Matrix‐Specific Aspects

For probiotic encapsulation, materials GRAS or with qualified presumption of safety (QPS) status are preferred for food applications. Sodium alginate, chitosan (approved in Japan, the European Union, and the United States as GRAS), gelatin, whey proteins, and inulin are widely accepted. However, novel polymers (e.g., plant mucilages, modified starches, synthetic polyelectrolytes) may require novel food authorization, particularly in the European Union, significantly delaying time to market. Chitosan, although effective for pH‐responsive release, carries regulatory restrictions in some jurisdictions regarding purity specifications and maximum daily intake.

Another point of concern is that encapsulation may inevitably affect food texture and mouthfeel. Large beads (>1 mm) produced by extrusion are noticeable in fluid products, such as beverages and yogurt drinks, but may be acceptable in solid matrices, including cheese and meat products. Emulsification and electrospraying generate small particles that are generally imperceptible to consumers, making them particularly suitable for beverage applications. In addition, lipid‐based coatings (e.g., those produced by emulsification) may impart a waxy or fatty mouthfeel, which can be undesirable in low‐fat products.

The choice of encapsulation strategy should follow a matrix‐driven decision process. In high‐acid fruit juices (pH 2.5–4.0, refrigerated shelf life ≤ 30 days), alginate or alginate‐chitosan beads (extrusion or emulsification) provide immediate pH protection, with emulsification preferred for clear juices to avoid sedimentation. In contrast, in high‐fat matrices (chocolate, cheese, ice cream, aw < 0.6), direct incorporation without encapsulation is often sufficient; small beads (< 50 µm) preserve sensory quality when additional protection is needed.

For infant formula powders, spray drying with dairy‐based carriers, such as whey protein and skim milk, remains the industrial standard for probiotic protection. In contrast, for fermented meat products, extrusion using alginate beads (1–2 mm) is often preferred, as the beads are effectively masked by the product's texture. For cereal bars and baked goods, spore‐forming *Bacillus* strains are generally preferred, as encapsulation is often secondary to strain selection. In the case of clear beverages, emulsification techniques that produce particles smaller than 100 µm are required to avoid sedimentation. Table 4 provides quantitative evidence supporting these recommendations.

## 6 New Generation of Probiotics Produced by Genetic Engineering for Food Application

Technological stresses during probiotic formulation and GI survival remain major challenges for industrial application (Bustos et al. 2024). Bioengineering offers opportunities to improve viability, functionality, and therapeutic potential through strategies aimed at reducing virulence factors, increasing stress resistance, and introducing novel beneficial traits. However, unlike pharmaceutical applications, genetic engineering for food purposes faces stringent regulatory hurdles that have, to date, prevented the commercial approval of any genetically modified probiotic as a food ingredient in major markets.

### 6.1 Food‐Relevant Engineering Strategies

Genetic engineering of probiotic bacteria for food applications focuses on three main areas: (i) enhancing stress tolerance to improve survival during processing and storage; (ii) optimizing metabolic pathways to enhance flavor, texture, or nutritional properties; and (iii) developing food‐grade vectors without antibiotic resistance markers.

#### 6.1.1 Stress Tolerance Enhancement

Stress tolerance has been enhanced through the successful implementation of several strategies aimed at improving microbial survival during food processing. The betaine uptake system BetL from *Listeria*, critical for salt tolerance, was expressed in *Limosilactobacillus salivarius* UCC118, improving resistance to osmotic, freezing, barometric, and chilling stress and enhancing survival during spray‐ and freeze‐drying (Sheehan et al. 2006). Incorporation of *betL* and *bilE* into *Bifidobacterium breve* UCC2003 increased survival in gastric juice and bile salts, improving GI persistence (Sheehan et al. 2007; Watson et al. 2008). Trehalose accumulation is a key cellular defense against acid, cold, and heat stress. Expression of the ostAB trehalose‐synthesis operon from *Escherichia coli* and a trehalose‐synthesis gene from *Propionibacterium freudenreichii* in *L. lactis* improved survival during freeze‐drying, bile resistance, and acid tolerance (Termont et al. 2006; Carvalho et al. 2011). For oxidative stress protection, which is particularly relevant for oxygen‐sensitive *Bifidobacterium* in fermented milk products, *B. longum* 105 A was genetically modified with the *katE* catalase gene from *B. subtilis*, which increased its aerobic survival (He et al. 2012). Co‐expression of *katL* (catalase from *L. plantarum*) and *sodA* (superoxide dismutase from *S. thermophilus*) in *B. longum* NCC2705 provided synergistic protection against ROS (Zuo et al. 2014).

#### 6.1.2 Metabolic Pathway Optimization

LAB are known to produce several biologically active metabolites, including GABA, EPSs, conjugated linoleic acid (CLA), and bacteriocins, which contribute to improvements in flavor, texture, and nutritional quality, while also enhancing the nutraceutical properties of final food products. For dairy applications, engineered *S. thermophilus* strains expressing enhanced proteolytic systems can accelerate processing and support the manufacture of reduced‐sugar foods, flavor enhancers, and texturizing agents (Markakiou et al. 2020; Plavec and Berlec 2020; Romero‐Luna et al. 2022). Milk proteins serve as substrates for the proteolytic systems of LAB and genetically modified probiotics, generating bioactive peptides with diverse functional properties (Hafeez et al. 2014). Modified probiotics can increase the production of specific bacterial enzymes with limited hydrolytic activity, thereby preventing excessive degradation of bioactive peptides and preserving their biological activity (Daliri et al. 2017; Chai et al. 2020).

#### 6.1.3 CRISPR–Cas9 and Food‐Grade Selection Markers

Recent advances have significantly expanded the toolkit for probiotic engineering through the exploitation of endogenous CRISPR‒Cas systems, particularly CRISPR–Cas9, enabling more precise and efficient genetic modifications in probiotic strains. Tang et al. (2026) developed a dual genome engineering strategy integrating CRISPR–Cas9‐mediated knock‐in with Cre/loxP‐driven genome reduction to streamline the genome of *L. lactis* NZ9000 and enable stable expression of a high‐activity uricase variant. The resulting strain demonstrated enhanced enzymatic performance in vitro and established a genetically stable and biosafe probiotic chassis with moderate colonization capacity in the murine gut. Z. Xie et al. (2026) successfully exploited the endogenous Type II‐A CRISPR‒Cas system for functional engineering of *L. rhamnosus* GG, one of the most extensively studied probiotic strains. Using a plasmid interference assay with single‐nucleotide substitutions, they confirmed the precise PAM requirement as 5′‐NGAAA‐3′ and developed a genome editing workflow enabling targeted deletions and insertions across multiple loci with practical editing efficiencies (11.1%–25.0%). These advances open possibilities for developing food‐grade engineered strains with enhanced processing robustness.

The examples above, particularly the expression of uricase (Tang et al. 2026), represent medically or veterinary‐oriented live biotherapeutics rather than food‐grade applications. While these demonstrate the potential of genetic engineering, they are not yet approved for food use. The lessons from these studies, particularly the successful use of CRISPR–Cas9 and food‐grade selection markers, are directly transferable to food applications. However, the regulatory pathway for food remains distinct and more stringent, as discussed previously.

A critical requirement for food applications is the elimination of antibiotic resistance markers, which has driven the development and use of food‐grade selection markers as safer alternatives for strain selection and genetic engineering. The *alr* gene, encoding alanine racemase (EC 5.1.1.1), has been successfully developed as a food‐grade selection marker in *L. lactis* and *L. plantarum* (Bron et al. 2002). Since isogenic mutants carrying an *alr* deletion (Δ*alr*) show auxotrophy for d‐alanine, plasmids carrying a heterologous *alr* can complement this auxotrophy, allowing stringent selection without antibiotics (Bron et al. 2002). Plasmids were stably maintained over 200 generations of culturing, demonstrating the feasibility of antibiotic‐free selection systems for industrial applications (Bron et al. 2002). The nisin‐controlled expression system, using the food‐grade inducer nisin, has also been widely adopted for controlled gene expression in LAB without antibiotics. Table 5 summarizes the engineering targets most relevant to food matrices, contrasting with the therapeutic proof‐of‐concept examples discussed above.

**TABLE 5:** Engineering targets for probiotic enhancement in food applications.

Target	Gene(s)/pathway	Engineered outcome	Food relevance	Key reference(s)
Oxidative stress tolerance	*katE* (catalase), *sodA* (superoxide dismutase)	Increased aerobic survival	Critical for oxygen‐sensitive *Bifidobacterium* in fermented milks	He et al. (2012), Zuo et al. (2014)
Bile/acid tolerance	*betL*, *bilE* (betaine uptake)	Enhanced survival in GI tract	Improves delivery to colon for all food matrices	Sheehan et al. (2006, 2007), Watson et al. (2008)
Heat/osmotic tolerance	*ostAB* (trehalose synthesis)	Improved survival during spray/freeze drying	Directly applicable to production of probiotic powders	Termont et al. (2006), Carvalho et al. (2011)
Exopolysaccharide production	*eps* gene clusters	Enhanced texture, cryoprotection	Improves mouthfeel and viability in frozen dairy	Plavec and Berlec (2020)
Flavor metabolite production	*als*, *ild* (diacetyl pathway)	Improved buttery/creamy aroma	Applicable to fermented dairy and plant‐based products	Markakiou et al. (2020)
Food‐grade selection markers	*alr* (alanine racemase)	Antibiotic‐free selection	Eliminates resistance genes, critical for regulatory approval	Bron et al. (2002)

*Note*: All listed references are cited elsewhere in this section. These targets are directly relevant to food applications; therapeutic targets (e.g., uricase expression) are discussed separately as proof‐of‐concept examples.

### 6.2 Regulatory Barriers for Engineered Probiotics in Food

Despite significant advances, no genetically modified probiotic has been approved for commercial use as a food product in the European Union, and only a handful have reached the US market under specific frameworks.

Under European Union regulations, genetically modified microorganisms intended for food use are subject to extensive safety testing (Salminen and Wright 2024). The EFSA QPS list, which provides a generic safety assessment pathway for microorganisms intentionally introduced into the food chain, currently excludes genetically modified microorganisms (Sanders et al. 2016; Salminen and Wright 2024). Any engineered probiotic must undergo the full novel food authorization process under Regulation (EU) 2015/2283, a pathway that can take 3–5 years and cost millions of euros (Sanders et al. 2016).

A critical distinction is between genome‐edited starter cultures for fermentation (e.g., *L. lactis*, *S. thermophilus* for cheese/yogurt) and engineered probiotics marketed as health interventions. Starter cultures are consumed as part of a fermented food, do not need to survive GI transit, and have a long safety history, thus facing lower regulatory hurdles. The European Union's QPS framework, however, does not distinguish between these use cases, excluding all GMMs regardless of application. For food applications, improved starter cultures may be a more immediately viable path for GM probiotics than standalone supplements requiring health claims.

In the United States, although the FDA's GRAS framework is considered less restrictive, most engineered probiotics are marketed as dietary supplements and regulated under the New Dietary Ingredient (NDI) notification framework rather than as conventional foods (Sanders et al. 2016). Live genetically modified probiotics face additional scrutiny; the FDA has issued warning letters against products making unsubstantiated health claims (Sanders et al. 2016). Furthermore, in 2023, the FDA issued warnings against probiotic products used to prevent necrotizing enterocolitis in infants following one infant death, underscoring the heightened safety scrutiny for live microbial products in vulnerable populations.

In practical terms, no genetically modified probiotic has yet been approved as a food additive or functional food ingredient in major global markets. The regulatory bottleneck, combined with consumer resistance driven by GMO labeling concerns, explains why, despite decades of research, nearly all commercial probiotic products still use nonengineered strains. Safety assessments of newly isolated strains require comprehensive genomic evaluation, including whole‐genome sequencing, virulence factor screening, and antibiotic resistance profiling (Salminen and Wright 2024; Grigore‐Gurgu et al. 2026). For modified probiotics, the bar is even higher, requiring evidence that modifications do not introduce pathogenicity or transferable resistance genes. Current evidence suggests that strains lacking mobile genetic elements, virulence factors, toxins, and antibiotic resistance genes, while possessing CRISPR‒Cas systems as beneficial safety features, represent the safest path forward (Najar et al. 2025).

### 6.3 Outlook

While genetic engineering offers powerful tools to enhance probiotic survival, stress tolerance, and metabolic capacity for food applications, regulatory hurdles and consumer acceptance remain substantial barriers to commercialization (Salminen and Wright 2024; Grigore‐Gurgu et al. 2026). The successful translation of engineered probiotics into food products will require (i) continued development of food‐grade vectors without antibiotic resistance markers (Bron et al. 2002); (ii) alignment with GRAS/QPS frameworks through comprehensive safety data (Sanders et al. 2016); (iii) transparent risk communication to address consumer concerns about GMOs; and (iv) demonstration of clear functional benefits that justify the additional regulatory burden.

Looking forward, genetic engineering could enable a paradigm shift from one‐size‐fits‐all probiotics to application‐specific designs through the co‐optimization of strain genotype and food matrix composition (Grigore‐Gurgu et al. 2026). In addition, strains engineered to produce protective metabolites in situ could create self‐protective microenvironments during processing and storage (Carvalho et al. 2011; Termont et al. 2006), simplifying formulations and appealing to clean‐label trends by reducing additive use. While still largely at the research stage, these approaches illustrate the long‐term potential of bioengineering to transform probiotic delivery (Salminen and Wright 2024).

For now, non‐GMO approaches such as stress adaptation, encapsulation, and postbiotic formulations remain more immediately viable for food applications.

## 7 Conclusion

The evidence reviewed here supports both hypotheses that guided this review. First, intrinsic matrix properties explain a substantial proportion of the observed variation in probiotic survival and functionality. The findings indicate that matrix characteristics, including pH, oxygen exposure, and buffering capacity, are key determinants of probiotic performance. While dairy products inherently provide protection, matrices with limited protective interactions, such as fruit juices (low pH) and cereal bars (low water activity), often require additional strategies to ensure probiotic survival and functionality.

Second, engineering approaches are most effective when integrated with matrix‐appropriate design rather than when applied in isolation. Quantitative comparisons (Table 3) demonstrate that the largest viability gains occur through combined strategies.

The strategies discussed in this review (strain selection, matrix design, encapsulation, prebiotics, and bioengineering) are not independent alternatives but complementary tools that should be selected based on the specific application. For example, a probiotic destined for a dairy product may rely primarily on the matrix's natural protection, while the same strain in a fruit juice would require encapsulation. Similarly, a stress‐sensitive strain may benefit from sublethal adaptation before processing, whereas a spore‐forming strain may not. For next‐generation probiotics with high oxygen sensitivity, a combination of encapsulation, prebiotic cryoprotectants, and genetic engineering may be necessary. Thus, the “from strain selection to bioengineering” journey is not a linear sequence but an integrated decision framework where each choice informs the next.

While recent reviews have comprehensively addressed specific domains of probiotic delivery encapsulation materials (Hussain et al. 2025; Yang et al. 2025), co‑encapsulation strategies (Ji et al. 2025), stress mechanisms (Bustos et al. 2024; Castro‑López et al. 2022), or food applications (Grujović et al. 2025; Nikitina and Khrundin 2026), no prior work has integrated these domains into a single end‑to‑end framework. This review provides a unique framework that integrates strain‐level selection (intrinsic tolerance and stress adaptation), matrix‐specific design, and encapsulation strategies; systematically compares probiotic performance across five major food matrices (dairy, fruits, cereals, chocolate and meat); clearly differentiates viability preservation from functional retention as distinct yet complementary objectives; and incorporates recent advances in CRISPR‐based genetic engineering alongside conventional probiotic protection strategies. By integrating these complementary approaches, this review provides a roadmap for selecting and combining strategies based on specific product requirements and target populations.

A critical insight is that viability and functionality are distinct attributes. Strains must not only maintain high cell counts but also retain functional characteristics such as stress tolerance, adhesion capacity, and metabolic activity. No single strategy ensures probiotic stability; optimal delivery requires integrating complementary approaches. At the strain level, intrinsic tolerance and stress adaptation provide robustness. At the matrix level, dairy offers natural protection, while nondairy matrices require technological support. At the delivery level, microencapsulation enables physical protection and targeted release. At the genetic level, CRISPR tools support next‐generation probiotics with enhanced stress tolerance and metabolic capacity for food applications. However, regulatory hurdles, particularly in the EU, where genetically modified microorganisms are excluded from the QPS list, and consumer acceptance challenges remain substantial barriers to commercialization. For now, non‐GMO approaches such as stress adaptation, encapsulation, and postbiotic formulations remain more immediately viable for food applications.

Key trends include (i) integration of omics and AI for strain‐matrix design with simultaneous assessment of viability and functionality; (ii) expansion of next‐generation probiotics via innovative encapsulation; (iii) application of bioengineering with robust biosafety assessments; and (iv) harmonization of regulatory frameworks recognizing the viability‐functionality distinction in efficacy assessment. Addressing these challenges will be essential to translate scientific advances into functional foods that deliver measurable health benefits.

As a narrative review, this work does not include systematic quantitative meta‐analysis, and the heterogeneity of experimental conditions across studies (strain specificity, matrix composition, processing parameters) limits direct comparability of outcomes. Most cited studies report in vitro or ex vivo data; human clinical trials validating these strategies under real‐world conditions remain limited. In addition, the review focuses primarily on traditional probiotic genera, with less emphasis on next‐generation probiotics due to the scarcity of food matrix studies. Despite these limitations, this review provides a comprehensive synthesis of current strategies and identifies key research gaps for future investigation.

## Author Contributions

**Sidney Rodrigues de Jesus Silva**: conceptualization, investigation, writing – original draft, methodology, validation, visualization, writing – review and editing, formal analysis, data curation. **Ramila Cristiane Rodrigues**: conceptualization, investigation, writing – original draft, methodology, validation, visualization, writing – review and editing, formal analysis, supervision, data curation. **Rosângela Freitas**: conceptualization, investigation, writing – original draft, methodology, validation, visualization, writing – review and editing, formal analysis, supervision, data curation. **Paulo César Stringheta**: supervision, writing – review and editing, writing – original draft, methodology, validation. **Pedro Henrique Campelo**: supervision, writing – review and editing, validation, methodology, writing – original draft, investigation, conceptualization. **Evandro Martins**: conceptualization, investigation, writing – original draft, methodology, validation, visualization, writing – review and editing, formal analysis, supervision, resources, data curation, project administration.

## Conflicts of Interest

The authors declare no conflicts of interest.
